# Integrated analysis identifies the IL6/JAK/STAT signaling pathway and the estrogen response pathway associated with the pathogenesis of intracranial aneurysms

**DOI:** 10.3389/fimmu.2022.1046765

**Published:** 2022-11-14

**Authors:** Aihong Wu, Chao Zhao, Shanling Mou, Shengjun Li, Xinchun Cui, Ronghua Zhang

**Affiliations:** ^1^ Library, Qufu Normal University, Rizhao, Shandong, China; ^2^ Department of Neurosurgery, The Affiliated Rizhao People´s Hospital of Jining Medical University, Rizhao, Shandong, China; ^3^ School of Computer Science, Qufu Normal University, Rizhao, Shandong, China; ^4^ Department of Laboratory, The Affiliated Rizhao People´s Hospital of Jining Medical University, Rizhao, Shandong, China

**Keywords:** intracranial aneurysm, GSVA, IL6/JAK/STAT signaling pathway, estrogen, immune microenviroment, WGCNA

## Abstract

**Objective:**

We intended to identify the potential key biomarker and pathways that correlated with infiltrating immune cells during the pathogenesis of intracranial aneurysms (IA), to develop a diagnostic model, and to predict therapeutic drugs.

**Methods:**

Three datasets containing intracranial aneurysm tissue samples and normal artery control samples from Gene Expression Omnibus (GEO) were included. Gene-set variation analysis(GSVA) and gene set enrichment analysis (GSEA) were conducted to find the significant differentially expressed pathways in IA formation. The least absolute shrinkage and selection operator (LASSO) regression and the multivariate logistic regression analysis were performed to identify the characteristic genes in the IL6/JAK/STAT signaling pathway (ISP) and the estrogen response pathway (ERP). A diagnostic model was constructed. xCell was used to identify immune cell types in IA pathogenesis. We used the weighted gene co-expression network analysis (WGCNA) algorithm to explore the correlations between the key modules and the four traits. Potential therapeutic drugs were investigated in Enrichr and Drugbank database.

**Results:**

The ISP is significant positively correlated with IA onset. The biological function of the ISP is positively correlated with that of the ERP, and is significantly associated with immune cells activities. *CSF2RB*, *FAS*, *IL6*, *PTPN1*, *STAT2*, *TGFB1* of the ISP gene set and *ALDH3A2, COX6C, IGSF1, KRT18, MICB, NPY1R* of the ERP gene set were proved to be the characteristic genes. The *STAT2* gene can be the potential biomarker of IA onset. The immune score of IA samples was significantly higher than the controls. The *STAT2* gene expression is associated with infiltration of immune cells. The WGCNA results were consistent with our finds. Acetaminophen can be a potential therapeutic drug for IA targeting *STAT2.*

**Conclusions:**

We identified that the ISP was one of the most significant positively correlated pathways in IA onset, and it was activated in this process concordant with the ERP and immune responses. Except for beneficial effects, complex and multiple roles of estrogen may be involved in IA formation. STAT2 could be a potential biomarker and a promising therapeutic target of IA pathogenesis.

## Introduction

Intracranial aneurysm (IA) has been a life-threatening disease by now. According to angiographic studies, the prevalence of intracranial aneurysms in the general population ranges from 2.0 to 6.0 percent (1, 2). The mortality after IA rupture is about 30%, with a disability rate of 36.7% in surviving patients (3). The current treatment options for IA, endovascular coiling and surgical clipping, are invasive and somewhat risky. The molecular mechanisms of pathogenesis of IAs remain poorly elucidated. Noninvasive treatments have not been identified and implemented in clinical practice. Clarification of the mechanisms may contribute to the early diagnosis of IA and the development of potential safe and effective drug treatments.

The clinical data reveal that familial history of IA, high blood pressure, cigarette smoking, alcohol consumption, and female sex have been identified as risk factors of IA formation (4). Females have a significantly higher incidence of IA than males, and it is even higher in familial cases of IA (5–7), whereas the majority of risk factors, such as cigarette smoking, hypertension, atherosclerosis, and alcohol consumption, are more prevalent in men (8–10). This evidence implies that female sex steroids such as estrogen may play an important role in IA onset.

The recruitment and infiltration of immune cells have been identified as a critical stage in the occurrence of IA (11). For example, infiltrating macrophage can impair intracranial arterial wall integrity and contribute to IA formation (12). The tumor necrosis factor α (TNF-α)/NF-κB pathway may be involved in the development of IAs (13).

In this study, we sought to find the most significant differentially expressed pathway (DEP) that was associated with IA formation *via* the gene set variation analysis (GSVA) algorithm, and explored its correlation with the estrogen response pathway. We discovered the characteristic genes in these two pathways and the infiltration pattern of immune cells. A diagnostic model was constructed to distinguish IAs from controls using bioinformatics analysis. Potential drugs of IA were also detected.

**Figure 1:**
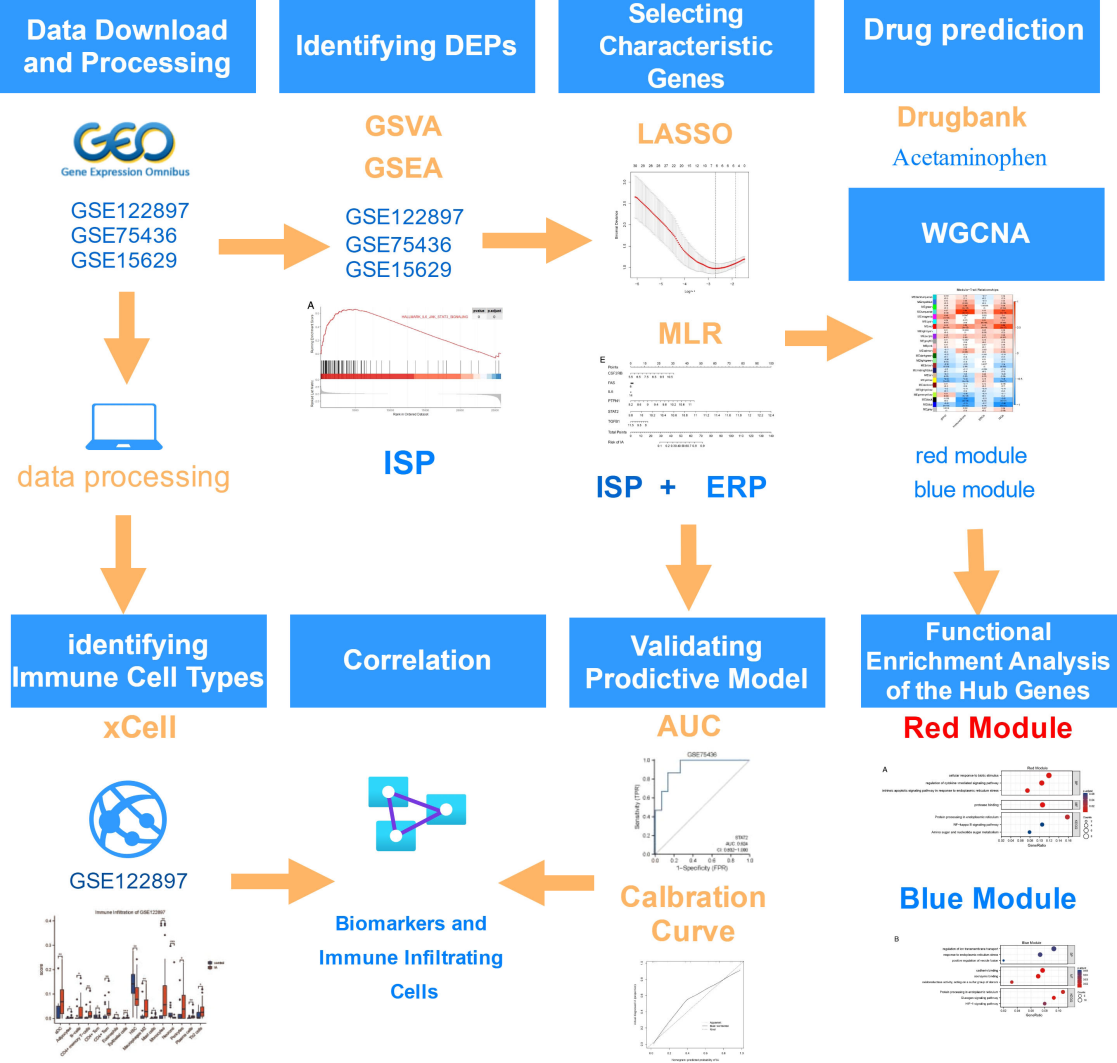
Flowchart on the construction and validation of the pathogenesis-related key biomarkers and pathways of intracranial aneurysm (IA) onset. GSVA, Gene Set Variation Analysis; GSEA, Gene Set Enrichment Analysis; ISP, IL6/JAK/STAT Signaling Pathway; ERP, Estrogen Response Pathway; LASSO, Least Absolute Shrinkage and Selection Operator; MLR, Multivariate Logistic Regression; AUC, Area Under the Curve; WGCNA, Weighted Gene Co-expression Network Analysis. This flowchart was generated and edited with the online free application, diagrams.net (https://app.diagrams.net/).

The workflow chart is **Figure 1**.

## Materials and methods

### Gene expression datasets and data processing

Three transcriptome-wide gene expression profiles of GSE122897, GSE75436 and GSE15629, were downloaded from Gene Expression Omnibus (GEO) (14). The RNA sequencing dataset GSE122897 contains 44 IA tissue samples and 16 control tissue samples (cerebral arteries), which was based on the GPL16791 (Illumina HiSeq 2500 Homo sapiens) platform. Two IA samples in GSE122897, GSM3487858 and GSM3487892, were rejected as their statuses were “unknown”. As an outlier, GSM3487895 was also omitted according to our previous research (15). A total of 41 IA samples and 16 control samples were included in the analysis. Raw read counts profile of GSE122897 was processed by “DESeq2” package for library normalization and independent filtering.

The microarray datasets GSE75436 included data from 15 IA tissues and 15 matched superficial temporal artery tissues of them and served as independently external validation. The microarray dataset GSE15629 provided a gene expression profile of 14 IA samples (8 ruptured and 6 unruptured) and 5 controls (middle meningeal arteries). The ruptured and unruptured IA samples were combined as IA tissue samples. All data used in the study were obtained from the GEO, and hence, ethics approval and informed consent were not required.

### Identification of the associated pathways with IA formation

We downloaded HALLMARK gene sets from the MSigDB database (http://www.gsea-msigdb.org/gsea/msigdb/index.jsp) to perform the GSVA algorithm using the R package “GSEABase”. The “h.all.v7.5.1.symbols.gmt” profile was used for this analysis. To reveal the enriched pathways within 50 HALLMARK gene sets, we used the “GSVA” package to evaluate enrichment score and assign pathway activity conditions between control and IA samples in GSE122897, GSE75436 and GSE15629 with a Gaussian distribution. Then, the limma package was also used to find the DEPs. Adjusted *p* value< 0.05 and |logFC|>0.3 were set as criteria. Gene set enrichment analysis (GSEA) was used to analyze the selected pathway activity in GSE122897 and GSE75436.

### Functional correlation analysis

The IL6/JAK/STAT signaling pathway gene set (M5897) and the estrogen response pathway gene sets (M5906 and M5907) were downloaded from the MSigDB database. The GSVA scores were used to investigate the relationship between the IL6/JAK/STAT signaling pathway (ISP) geneset and the estrogen response pathway (ERP) geneset based on expression profile data of GSE122897 and GSE75436. Gene Ontology (GO) analysis was performed by R package “clusterProfiler”. The significantly different Biological Process (BP) and Molecular function (MF) terms were screened with the criterion adjusted *p* value <0.05. The results were visualized by R package “enrichplot” and “ggplot2”.

### Selection of characteristic genes in ISP and ERP

The least absolute shrinkage and selection operator (LASSO) regression was performed to identify the characteristic genes with the highest IA predictive values of ISP and ERP based on expression profile data of GSE122897. Moreover, the predictive ability was further evaluated by multivariate logistic regression analysis with “rms” package using GSE122897 dataset.

### Validation of predictive models

The receiver operator characteristic (ROC) analyses were used to evaluate the capability of the characteristic genes to distinguish IA patients with controls using the “pROC” package based on GSE75436 dataset. The ROC curves were plotted by the “ggplot2” package, and the area under the curve (AUC) was also calculated. The calibration curve was conducted to analyze the accuracy of the predictive model during IA occurrence. Nomogram was established to illustrate the results of multivariate logistic analyses for the characteristic genes in ISP using the “rms” package.

### Correlation analysis between characteristic genes

Spearman correlation analysis was performed to explore the correlation between *STAT2* and the characteristic genes of the estrogen response pathway in GSE122897 and GSE75436.

### Correlation analysis between biomarker and immune cell infiltrations

xCell (16) was implemented to analyze the infiltration levels of immune cells on the basis of the expression profiling of GSE122897, and portrayals of cellular heterogeneity landscape for IA tissue expression profiles were acquired. Cutoff for significance were *p* value < 0.05. Correlation matrix of immune cells and the characteristic genes in ISP were constructed by The SangerBox 3.0 (http://vip.sangerbox.com/home.html).

### Construction of weighted gene co-expression network

We used the whole gene transcriptome expression profile of GSE122897 to perform weighted gene co-expression network analysis (WGCNA). Topology network of WGCNA was generated by “WGCNA” R package (17). To transform the adjacency matrix to a topological overlap matrix, the soft-threshold power (β) was eight when 0.85 was chosen as the correlation coefficient threshold with a scale-free R^2^ above 0.85 and a slope near 1. The minimum number of genes in modules was 80, and the cut height threshold was 0.3 for module detection. We produced a module-trait relationships chart and searched for the key modules that were closely related to group (IA or controls), immuneScore, ERP and ISP. The correlations among the eigengenes of the modules and the four traits were used to identify key modules.

We obtained the module significance (MS) resulted from calculating the average absolute gene significance (GS) of all the genes involved in the module. In the WGCNA, a module with high correlation score and *p* value <0.05 among all modules is defined as the key one and employed for further analysis.

### Functional enrichment analysis

Genes in the key module of group indicated a significant correlation with traits of IA occurrence. Hub genes in WGCNA were defined as those with a gene significance (|GS|) >0.4 and a module membership (|MM|) >0.8. Hub genes were selected to perform GO and Kyoto Encyclopedia of Genes and Genomes (KEGG) analyses. GO and KEGG pathway analyses were conducted to explore their biological functions utilizing the R package “ clusterProfiler “ (18). GO terms and KEGG pathways were explored with adjusted *p* value <0.05 were set as the cutoff and were visualized by the R package “GOplot” (19).

### Screening potential therapeutic drugs

The Enrichr (https://amp.pharm.mssm.edu/Enrichr/) and Drugbank platform (https://go.drugbank.com/) were used for drug screening of STAT2. Access of the DSigDB database was acquired using Enrichr. Potential therapeutic agents were determined by the adj. *p* values and the abundance of acting on STAT2 and co-expression genes. All experimentally validated drug-target information is displayed in Drugbank. We selected the drugs that can downregulate the expression of *STAT2* mRNA in it.

### Statistical analysis

The Wilcoxon test was applied to compare the difference of continuous variables between the two groups. All analyses were conducted using R4.1.0. software. The *p* value or adjusted *p* value< 0.05 were considered statistically significant. R packages of limma, WGCNA, ggplot2, export, clusterProfiler, rms, GSEABase, GSVA, ROCR, etc. were used in this study.

## Results

### Associated pathways with IA formation

**Figure 2:**
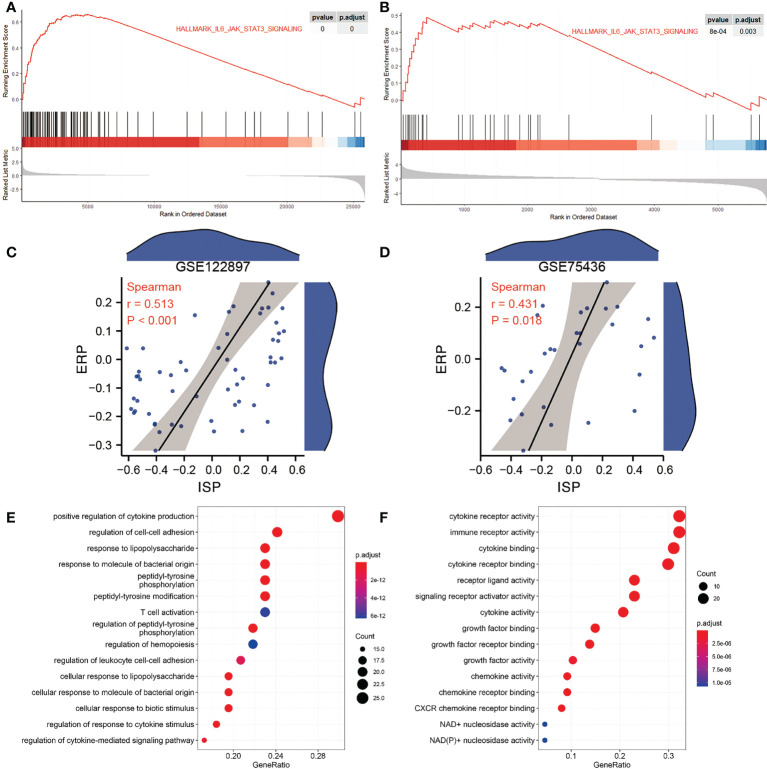
Functional Correlation Analysis of the IL6/JAK/STAT signaling pathway (ISP) **(A)** The Gene set enrichment analysis (GSEA) result of the ISP in GSE122897. **(B)** The GSEA result of the ISP in GSE75436. **(C)** The correlation of the ISP and the estrogen response pathway (ERP) in GSE122897. **(D)** The correlation of the ISP and ERP in GSE75436. **(E)** Gene Ontology (GO) Biological Process (BP) enrichment analysis. **(F)** GO Molecular function (MF) enrichment analysis.

The GSVA was conducted to analyze the significant DEPs between IAs and controls. The geneset with logFC >0.3 or logFC<-0.3 was defined accordingly as an upregulated or downregulated geneset of the three datasets (**Supplementary Table 1**). Three common pathways were significant positively correlated with IA onset according to GSVA results, which are HALLMARK_ALLOGRAFT_REJECTION, HALLMARK_INFLAMMATORY_RESPONSE and HALLMARK_IL6_JAK_STAT3_SIGNALING. We selected the IL6/JAK/STAT signaling geneset in our research as it has been mentioned in our previous work (15). In GSE122897 and GSE75436, the ISP is activated by GSEA. (**Figures 2A, B**)

### Functional correlation analysis

The GSVA is also performed to quantify the ISP and ERP scores of all samples. The ISP is positively correlated with the ERP in GSE122897 and GSE75436, as is shown in **Figures 2C, D**. To explore the physiological and pathological function of ISP, GO BP and GO MF enrichment analysis were performed with the GSE122897 dataset. The results manifested the ISP were mainly related to biological processes of immune cells, such as T cell activation, leukocyte adhesion, response to molecule of bacterial origin, response to biotic stimulus, regulation of response to cytokine stimulus, regulation of cytokine-mediated signaling pathway; molecular functions of immune response, such as cytokine and immune receptor activity, cytokine and its receptor binding, cytokine and chemokine activity, chemokine and CXCR chemokine receptor binding. (**Figures 2E, F**). These findings show that the function of ISP is significantly associated with immune cells activities.

### Selection of characteristic genes in ISP and ERP

In order to investigate the characteristic genes of the ISP and the ERP in IA formation, we obtained 87 genes of ISP and 299 genes of ERP from MSidDB. These two gene sets were used for LASSO regression with gene expression data of GSE122897 to select the valuable predictive genes.

**Figure 3:**
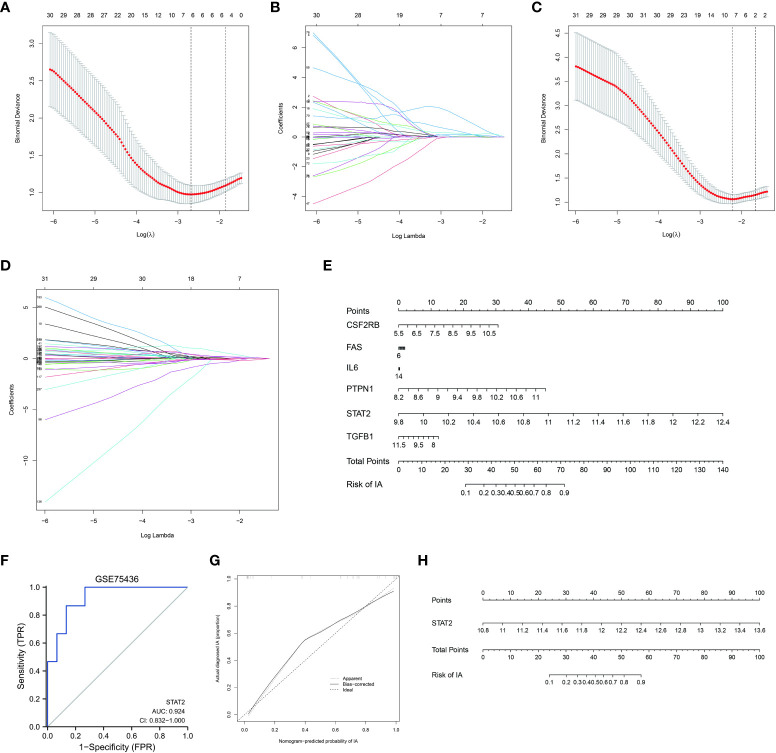
Selecting and validating characteristic genes **(A, B)** Investigation of the characteristic genes of the IL6/JAK/STAT signaling pathway (ISP) by the least absolute shrinkage and selection operator (LASSO) regression. **(C, D)** Investigation of the characteristic genes of the estrogen response pathway (ERP) by LASSO regression. **(E)** Nomogram based on the characteristic genes of ISP. **(F)** Validation of the diagnostic efficacy of STAT2 for intracranial aneurysm (IA) by the receiver operator characteristic (ROC)analysis. **(G)** Predictive accuracy of STAT2 for IA by the calibration curve. **(H)** Nomogram based on STAT2 as diagnostic tool for IA formation.

Firstly, we retained 6 genes within ISP with lambda = 0.0681, including *CSF2RB*, *FAS*, *IL6*, *PTPN1*, *STAT2*, *TGFB1* (**Figures 3A, B**). Multivariate logistic regression was performed to further identify the pivotal genes to simplify the model. The *p* value of *STAT2* was <0.05 in the logistic regression model, implying that *STAT2* has a greater contribution to the model than the other genes and might be an independent risk factor of IA occurrence (**Supplementary Table 2**). The expression levels of *STAT2* were significantly increased in IA samples compared with control samples in GSE122897 and GSE75436 (**Supplementary Image 1A, 1B**). Therefore, we constructed a diagnostic model based on *STAT2.*

Next, we obtained 7 genes within ERP with lambda = 0.124, including *ALDH3A2, COX6C, GAB2, IGSF1, KRT18, MICB, NPY1R* (**Figures 3C, D**). Multivariate logistic regression was also performed; whereas, the *p* value of each gene was >0.05 in this logistic regression model (**Supplementary Table 3**). The expression level of *GAB2* was not significantly different between IAs and controls in GSE75436 and it was omitted in this model. The expression patterns of the other six characteristic genes were the same in GSE122897 as in GSE75436 (**Supplementary Image 1C, 1D**).

### Diagnostic efficacy and validation of the diagnostic model of IA formation

Nomogram based on the characteristic genes of ISP was constructed as diagnostic tools for IA formation. In the nomogram, each characteristic gene corresponded to a score, and the total score was calculated by adding the scores for all characteristic genes. The total points corresponded to different risks of IA onset. (**Figure 3G**)

The ROC curves were plotted and the AUC was calculated to distinguish IA samples from controls. The AUC of *STAT2* were greater than 0.8 in GSE75436 (**Figure 3E**). The AUCs of *ALDH3A2, COX6C, IGSF1, KRT18, MICB, NPY1R* (**Supplementary Image 2A**) and the combination of them (**Supplementary Image 2B**) were greater than 0.7 in GSE75436. These results indicated a moderate diagnostic efficacy of these genes as new biomarkers.

The calibration curve demonstrated that the predictive model of *STAT2* enabled an accurate estimation during the progression of IA occurrence (**Figure 3F**). Nomogram was shown in **Figure 3H**.These analyses indicated that the *STAT2* gene can predict the onset of IA.

### Correlation analysis between characteristic genes

**Figure 4:**
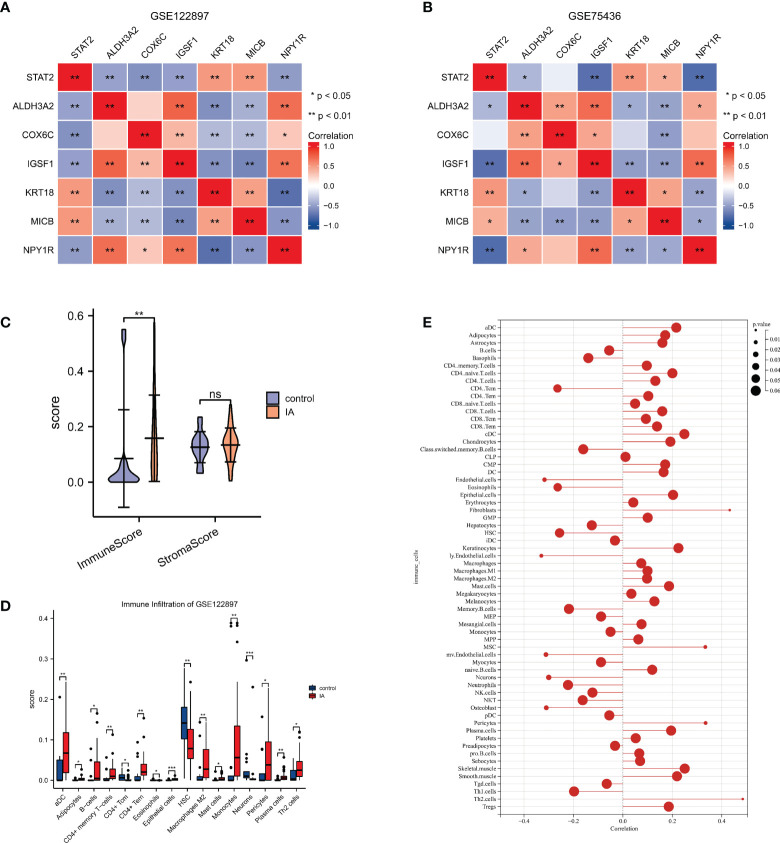
The correlations of *STAT2*, the estrogen response pathway (ERP) and the immune infiltrating cells **(A, B)** The correlations of *STAT2* and the characteristic genes of the ERP in GSE122897 and GSE75436. **(C)** The immune score and the stromal score of intracranial aneurysm (IA) samples compared with controls. **(D)** The infiltrating cells of IA samples compared with controls. **(E)** The correlation of *STAT2* and the infiltrating cells. ns=no significance, *p<0.05, **p<0.01, ***p<0.001.

Our research displayed that *KRT18, MICB* were positively correlated with *STAT2* (*p* value <0.05), and *ALDH3A2*, *IGSF1*, *NPY1R* were negatively correlated with *STAT2* (*p* value <0.05) in GSE122897 and in GSE75436; whereas *COX6C* did not show significant correlation with *STAT2* in GSE75436. (**Figures 4A, B**)

### Correlation analysis between biomarker and immune cell infiltrations

xCell was used to identify immune and stromal cell types that could play roles in IA pathogenesis. Using the GSE122897 expression data, xCell generated enrichment scores for various cell types that were shown in (**Supplementary Image 2C**). The immune score of IA samples was significantly higher than the controls, while there were no significant differences between the stromal scores of IA tissues and controls (**Figure 4C**). This result indicated that the immune microenvironment played a more important role in IA formation than the stromal microenvironment. There were 16 cell types with significant differences in IA tissues vs. controls in the GSE122897 dataset as shown in **Figure 4D**. The correlation of *STAT2* and infiltrating cells was recorded in **Supplementary Data Sheet 1** and visualized in **Figure 4E**. The scores of lymphatic (ly) endothelial cells, endothelial cells, microvascular (mv) endothelial cells, osteoblasts, neurons, CD4 central memory T cells (Tcm) and eosinophils were significantly decreased, while T-helper cells 2 (Th2 cells), fibroblasts, pericytes and mesenchymal stem cells were significantly enriched.

### Gene co-expression networks

**Figure 5:**
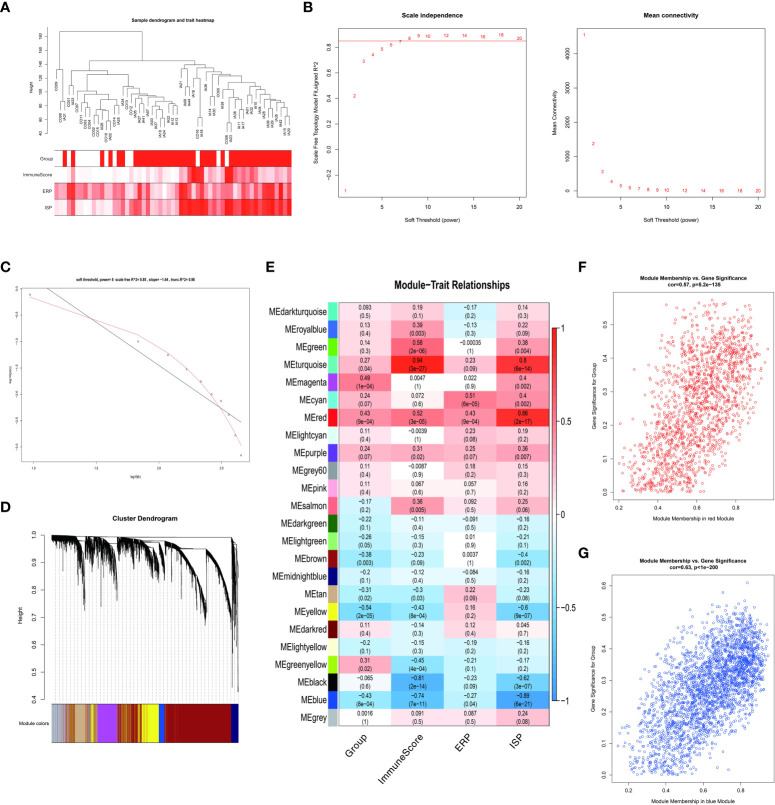
Weighted gene co-expression network analysis **(A)** Clustering dendrogram of total genes related to intracranial aneurysm (IA) onset. **(B, C)** Softthreshold power analysis implemented to obtain the scale-free fit index of the network topology. **(D)** Identification of modules. **(E)** Heatmap shows the relationships between different modules and traits. **(F)** Scatter plot of module eigengenes in the red module. **(G)** Scatter plot of module eigengenes in the blue module.

In this study, the cohort of GSE122897 consisted of 41 IA tissues and 16 cerebral artery controls. The sample clustering dendrograms of four traits (group, immuneScore, ERP and ISP) were shown in **Figure 5A**. In the WGCNA, we correlated each module with traits in the GSE122897 dataset by calculating the MS for each module-trait correlation. When 0.85 was used as the correlation coefficient threshold, the soft power β was selected as 8 (**Figures 5B, D**). Twenty-three co-expression modules were constructed by using the average linkage hierarchical clustering algorithm (**Figure 5E**). The red module had significant positive correlations with the four traits, whereas the blue module had significant negative correlations with these traits (**Figure 5C**). These results indicated strong associations among these traits. Seventy-six genes in the red module were identified as hub genes based on the cut-off criteria (|MM| > 0.8 and |GS| > 0.4) (**Supplementary Data Sheet 2**) (**Figure 5F**). In the blue module, 171 hub genes were identified (**Supplementary Data Sheet 3**) (**Figure 5G**).

### Functional enrichment analysis of the hub genes

**Figure 6:**
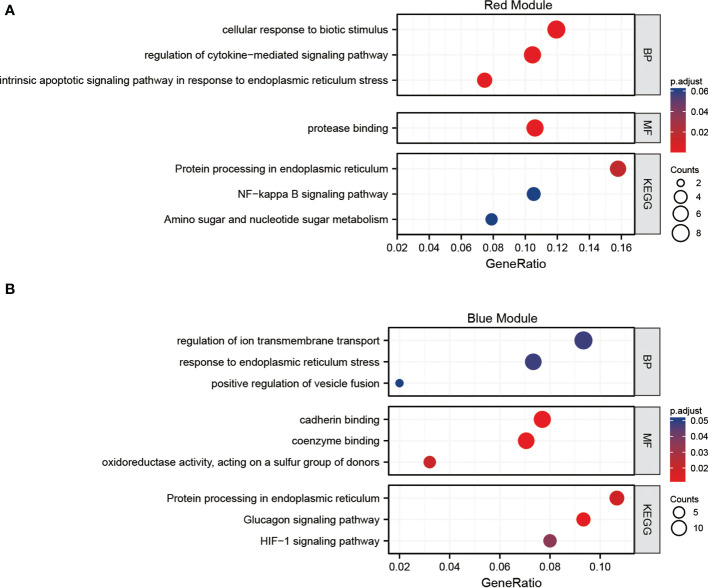
Functional analysis **(A)** Gene Ontology (GO) and Kyoto Encyclopedia of Genes and Genomes (KEGG) pathways enrichment of the red module of the weighted gene co-expression network analysis (WGCNA). **(B)** GO and KEGG pathways enrichment of the blue module of the WGCNA.

The GO BP enrichment analysis showed that the hub genes in the red module were mainly enriched in cellular response to biotic stimulus, regulation of cytokine-mediated signaling pathway, intrinsic apoptotic signaling pathway in response to endoplasmic reticulum stress, etc. MF enrichment analysis showed that these genes play an important role in protease binding. Protein processing in endoplasmic reticulum is the only pathway resulted from KEGG analysis (adj. *p* value <0.05). (**Figure 6A**)

The GO BP enrichment analysis revealed that the hub genes in the blue module were mainly enriched in response to endoplasmic reticulum stress, regulation of ion transmembrane transport. MF enrichment analysis showed that these genes play an important role in cadherin and coenzyme binding. Protein processing in endoplasmic reticulum and HIF-1 signaling pathway are enriched in KEGG analysis (adj. *p* value <0.05). (**Figure 6B**). The functions of hub genes in the red module and blue module are related to cellular substance metabolism.

### Screening of potential therapeutic drugs

Enrichr platform analysis found that suloctidil HL60 UP, prenylamine HL60 UP, terfenadine HL60 UP and acetaminophen CTD 00005295 etc. have been identified as drugs that regulate action for both *STAT2* and co-expression genes (**Supplementary Data Sheet 4**). Acetaminophen was approved to downregulate the expression of *STAT2* mRNA in Drugbank database (**Supplementary Data Sheet 5**). Acetaminophen can be a potential therapeutic drug for IA targeting *STAT2.*

## Discussion

The activation of ISP can stimulate cell proliferation, differentiation, migration and apoptosis in various diseases, e.g., cardiovascular diseases, colorectal cancer and myelofibrosis (20–22). It is well established that the ISP acts as a critical regulator of inflammatory processes during the pathogenesis of atherosclerosis and arterial hypertension, and its members are constitutively expressed in the vessel wall and transfer intracellular signaling events of various receptor families, e.g., that of cytokines, growth factors and vasoactive peptides (23). However, no study has reported the role of ISP in IA onset so far. Previously, we found that the ISP correlated with *SLC2A12*, a potential biomarker in IA occurrence (15). In the present study, the role of ISP in IA formation was explored for the first time using bioinformatics analysis. It was significantly activated in IA formation by GSEA, and is significantly associated with immune cells activities. Then, we constructed a one-gene signature of *STAT2* and generated the correlation networks of *STAT2* with characteristic genes of ERP and infiltrating immune cells.

In this research, we found the ERP was activated in IA formation together with the ISP and immune response. But the roles of estrogen in IA formation remain controversial nowadays. Generally, the majority of present studies suggest a beneficial effect of estrogen in IA pathogenesis (24, 25). In a rat model of IA, the incidence of IA is significantly higher in ovariectomized females than in males (24). A clinical meta-analysis study conducted by Vlak MH, et al. found that the female/male prevalence ratio (PR) of IA was 1.1 in populations with mean age of 50 years old, while this PR went up to 2.2 in those older than 50 years old (25). The decline of estrogen concentration in peri- and post-menopause periods can favor the formation of IAs.

However, it is well known that women are predisposed to IA (26). Female gender is an independent risk factor for the formation of new IAs (8). The preponderance of IAs between women and men starts after the first two decades of life (25). The role of female sex steroids such as estrogen may contribute to the sex disparities that exist in the presentation of IA. Moreover, estrogen can enhance immune response resulting in many acquired immunity and autoimmune diseases (27). Except for the beneficial effect of estrogen, the complex and multiple roles of estrogen may be involved in IA formation. In our research, the ISP, an immune-related pathway, was activated in IA formation, and the ERP was positively correlated with the ISP. Then we deduced that the ERP was positively correlated to this progress.

In addition, the WGCNA results were consistent with the above findings. The four traits, group, immuneScore, ERP and ISP, shared the same red module in WGCNA with significant positive correlations and the same blue module in WGCNA with significant negative correlations.

Vascular inflammation/immune responses were involved in IA formation, with neutrophil and macrophage infiltration, oxidative stress, fragmentation of the internal elastic lamina, and degradation of the extracellular matrix by metalloproteinase (28, 29). Estrogen is implicated in immune responses (30). The estrogen receptors (ERs) are expressed by many immunological cells including T cells, B cells, macrophages, and dendritic cells (DCs), suggesting a direct effect of estrogens on immune responses. Estrogen and ERs can alleviate the inflammation of arterial cell wall matrix and apoptotic pathways (31–33). G-protein coupled estrogen receptor (GPER) is a member of ERs, and it can induce apoptosis of vascular endothelial cells (34). The dysfunction of vascular endothelial cells is the initial part of IA formation (35, 36). Whereas, the other two members of ERs, ERα and ERβ, show a protective effect on IA (37). We speculate that there is a functional balance between ERs during the initiation of IA. Our research can shed light on the renewed understanding of the roles of estrogen involved in IA formation.

Studies manifested that estrogen deficiency leads to endothelial dysfunction and inflammation (38). And then, hemodynamic stress on the endothelial cells of the cerebral arteries triggers the pathogenesis of IAs, causing prolonged and excess inflammation in the vessel wall (39). The inflammation cellular infiltrations were discovered to occur in the IA fundus in 1966 (40). Since then, a series of experimental reports and animal studies have supported the role of inflammation in the pathogenesis of IA. The infiltration of immune cells including macrophages, T cells and Mast cells is a characteristic of IA (41–43). Cytokines are powerful mediators of the immune response and are reported to be involved in the pathogenesis of IA, especially IL1β, IL6, and tumor necrosis factor-α (TNFα) (44–46). Our research indicated that the immune microenvironment played a more important role in IA formation than the stromal microenvironment. Infiltrating immune cells included the B cells, CD4+ memory T cells, macrophages, monocytes, Th2 cells. These findings corroborated the previous researches.

To the best of our knowledge, the function of *STAT2* in IA pathogenesis has not been studied by now. We identified that *STAT2* was a potential biomarker with moderate diagnostic efficacy and an accurate estimation to distinguish IA samples from normal controls. *STAT2* may be a promising therapeutic target for the prevention of IA pathogenesis. Acetaminophen was approved to a potential drug targeting *STAT2* in Drugbank database. Nonsteroidal anti-inflammatory drugs (NSAIDs) are currently being investigated as a potential pharmaceutical treatment for patients with IA (47). Hasan DM reported acetylsalicylic acid, a member of NSAIDs, can attenuate inflammation in the walls of human cerebral aneurysms (48). More pharmacological research in this field will pave the way for the prevention and treatment of IA.

Limitations exist in this study. First, no experimental validation was performed in our research. Obtaining test samples (aneurysm domes) are getting more challenging at this stage. Compared with the microsurgical clipping, the endovascular coiling of IAs (ruptured and unruptured) shows more positive outcomes (49, 50). The endovascular treatment has become popular for the treatment of IAs and continues to take momentum around the world. Even in the open operation of IA, the key point is to clip the neck of IA but not to remove the dome of it (obtaining test samples). More exposure of microsurgical view may lead to more complications. Obtaining biological samples need more time and multicenter cooperation. We think animal models with endogenously induced IA formation will supplement reliable data in the future (15). Second, the control samples in this study included external carotid arteries. External and internal carotid arteries may differ at the transcriptome level. Third, the estrogen response pathway gene set was a combination of M5906 (early estrogen response) and M5907 (late estrogen response) from the MSigDB database. This combination may cause variations in the results.

## Conclusions

In the present study we identified that the ISP was one of the most significant positively correlated pathways with IA onset. The ISP, ERP and immune response were all activated together in this process. Except for the beneficial effect, complex and multiple roles of estrogen may be involved in IA formation. STAT2 could be a potential biomarker and a promising therapeutic target for the pathogenesis of IA.

## Data availability statement

The original contributions presented in the study are included in the article/**Supplementary Material.** Further inquiries can be directed to the corresponding author.

## Author contributions

CZ designed the study. SL, XC and RZ performed the data analyses. AW and SM wrote the manuscript. CZ revised the manuscript. All authors contributed to the article and approved the submitted version.

## Funding

This study was supported by the Key Research and Development Program of Rizhao City (2021ZDYF020205).

## Acknowledgments

We especially thank Dr. Shipeng Guo (Department of Endocrine Breast Surgery, The First Affiliated Hospital of Chongqing Medical University, Chongqing, China) for his guidance on bioinformatics and conceptualization.

## Conflict of interest

The authors declare that the research was conducted in the absence of any commercial or financial relationships that could be construed as a potential conflict of interest.

## Publisher’s note

All claims expressed in this article are solely those of the authors and do not necessarily represent those of their affiliated organizations, or those of the publisher, the editors and the reviewers. Any product that may be evaluated in this article, or claim that may be made by its manufacturer, is not guaranteed or endorsed by the publisher.

## Supplementary material

Supplementary Figure 1 Expression patterns of characteristic genes **(A)** The expression levels of *STAT2* in GSE122897. **(B)** The expression levels of *STAT2* in GSE75436. **(C)** The expression patterns of the characteristic genes of the estrogen response pathway (ERP) in GSE122897. **(D)** The expression patterns of the characteristic genes of the ERP in GSE75436.

Supplementary Figure 2 Discrimination ability of the six characteristic genes of the estrogen response pathway (ERP) and cell type enrichment result from xCell **(A)** The AUCs of the six characteristic genes of the ERP in GSE75436. **(B)** The AUC of the combination of the six characteristic genes of the ERP in GSE75436 **(C)** Cell type enrichment result from xCell.

## Abbreviations

IA, Intracranial Aneurysm; DEP, differentially expressed pathway; GSVA, Gene Set Variation Analysis; GSEA, Gene Set Enrichment Analysis; ISP, IL6/JAK/STAT Signaling Pathway; ERP, Estrogen Response Pathway; GO, Gene Ontology; BP, Biological Process; MF, Molecular Function; KEGG, Kyoto Encyclopedia of Genes and Genomes; LR, LASSO Regression; MLR, Multivariate Logistic Regression; ROC, Receiver Operator Characteristic; WGCNA, Weighted Gene Co-expression Network Analysis; NSAID, Nonsteroidal Anti-inflammatory Drug.
