# Supplementary figures and images for: Integrated analysis identifies the IL6/JAK/STAT signaling pathway and the estrogen response pathway associated with the pathogenesis of intracranial aneurysms
Source: Front Immunol. 2022 Nov 14;13:1046765. doi: 10.3389/fimmu.2022.1046765 (PMC9702531; doi:10.3389/fimmu.2022.1046765)

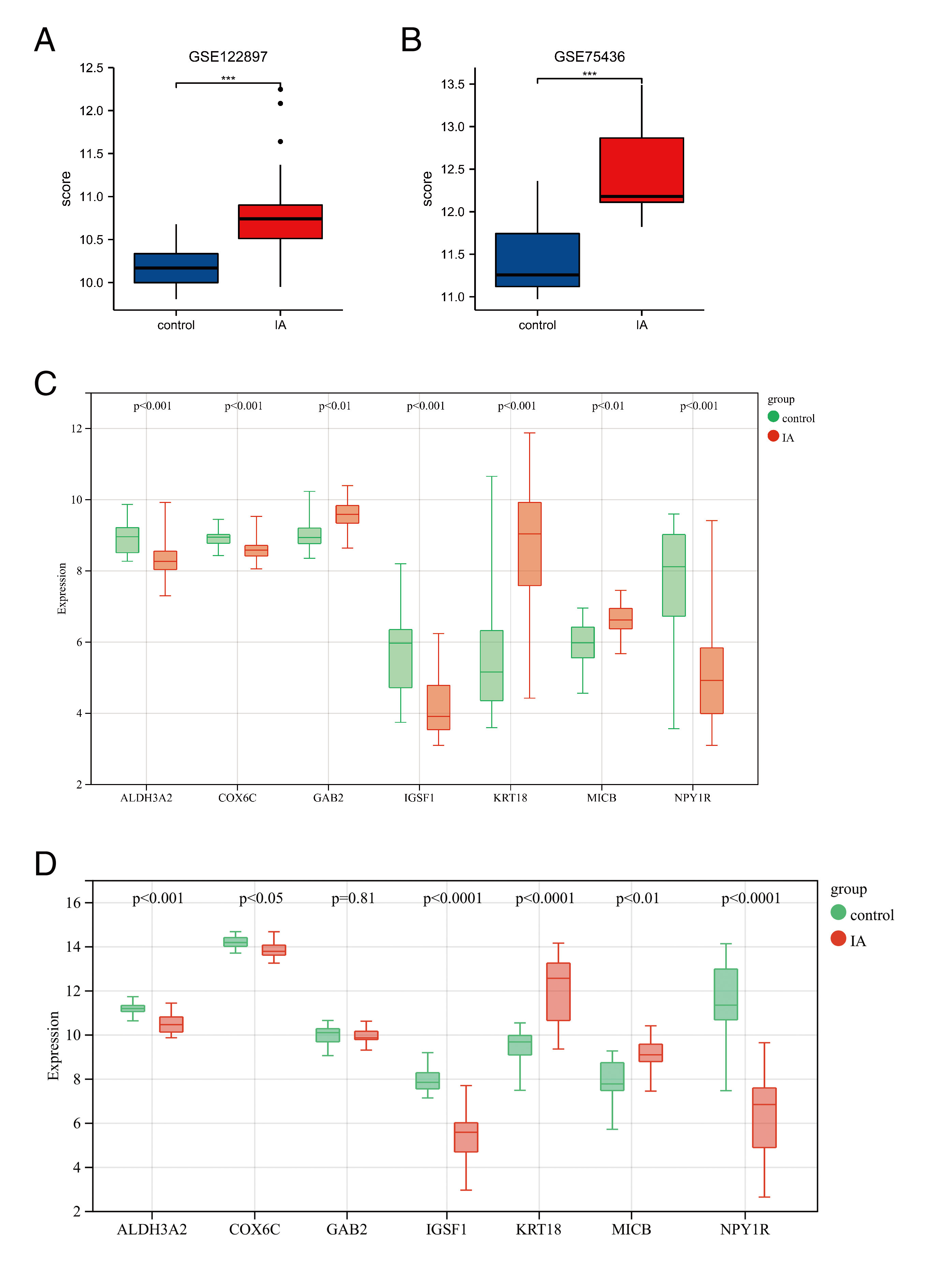

Supplement: Supplementary Figure 1 — Expression patterns of characteristic genes (A) The expression levels of STAT2 in GSE122897. (B) The expression levels of STAT2 in GSE75436. (C) The expression patterns of the characteristic genes of the estrogen response pathway (ERP) in GSE122897. (D) The expression patterns of the characteristic genes of the ERP in GSE75436. [file Image_1.jpeg]

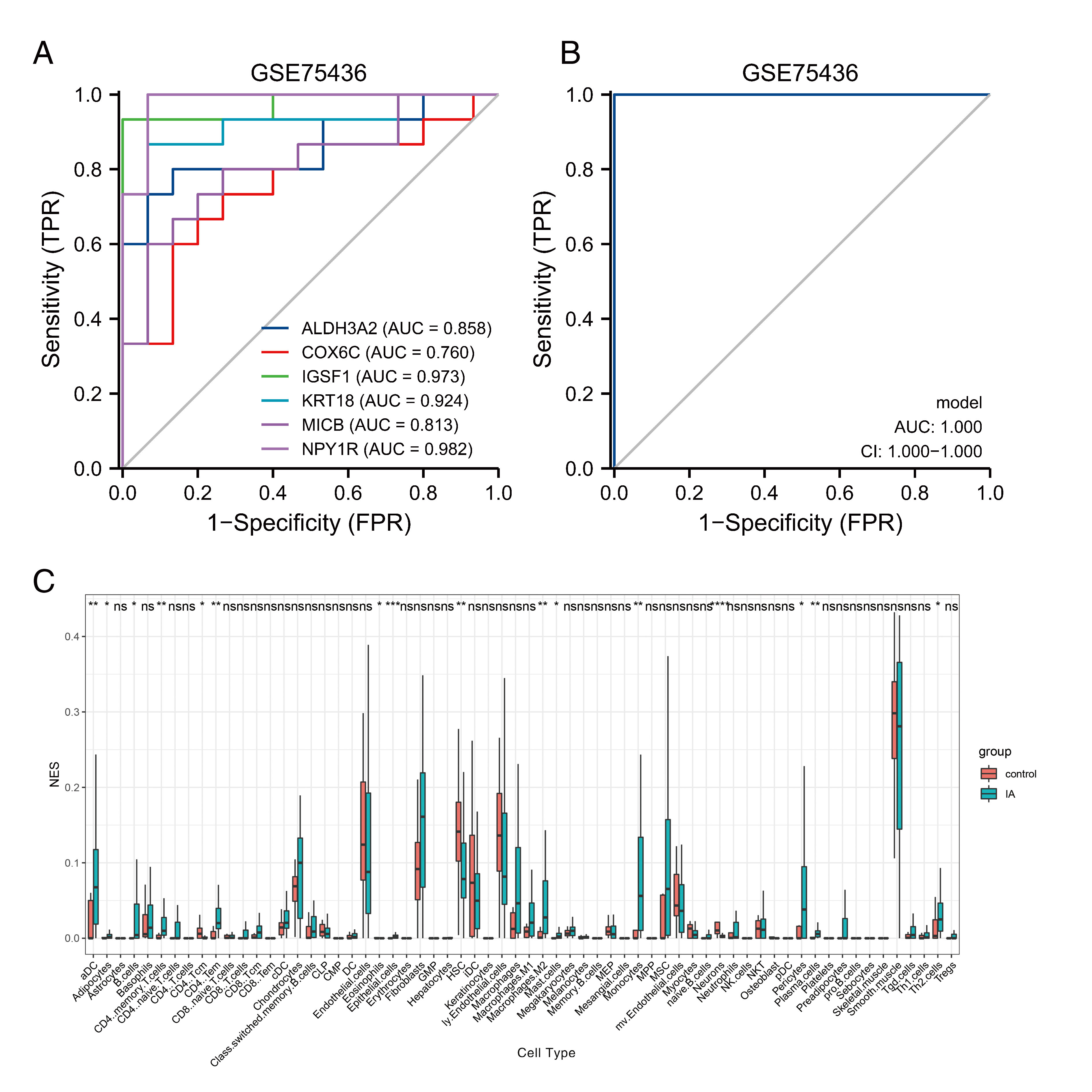

Supplement: Supplementary Figure 2 — Discrimination ability of the six characteristic genes of the estrogen response pathway (ERP) and cell type enrichment result from xCell (A) The AUCs of the six characteristic genes of the ERP in GSE75436. (B) The AUC of the combination of the six characteristic genes of the ERP in GSE75436 (C) Cell type enrichment result from xCell. [file Image_2.jpeg]
